# Mental healthcare planning: current and future challenges

**DOI:** 10.1007/s40211-026-00577-3

**Published:** 2026-04-29

**Authors:** Johannes Wancata, Fabian Friedrich

**Affiliations:** Clinical Division of Social Psychiatry, Department of Psychiatry and Psychotherapy, Medical University of Vienna, Währinger Gürtel 18–20, 1090 Vienna, Austria

**Keywords:** Psychiatric services, Psychiatry reform, De-institutionalization, Community treatment, Comorbidity, Psychiatrische Einrichtungen, Psychiatriereform, Deinstitutionalisierung, Gemeindenahe Versorgung, Komorbidität

## Abstract

Reforms of psychiatric care systems in Western countries have been guided by several principles such as de-institutionalization, mental healthcare close to the community, and integration into general healthcare. This paper reflects the achievements, shortfalls, and problems of selected aspects of mental health reforms. Despite major improvements, there have also been some setbacks. We consider such analyses to be essential for future care planning.

## Abstract (de)

Die Reformen der psychiatrischen Versorgung in den westlichen Ländern waren von einigen grundlegenden Prinzipien geleitet. Zu diesen gehörten beispielsweise Deinstitutionalisierung, Gemeindenähe der psychiatrischen Versorgung und eine Integration in die allgemeine Gesundheitsversorgung. Im vorliegenden Artikel werden Erfolge, Mängel und Probleme einiger ausgewählter Bereiche der Psychiatriereformen betrachtet. Neben großen Verbesserungen gab es aber auch einige Rückschritte. Wir denken, dass solche Analysen für eine Weiterentwicklung der Versorgungsplanung essenziell sind.

## Introduction

Until the 1970s, persons with severe and chronic mental illness often spent for long periods in old-fashioned psychiatric hospitals. In these hospitals, seclusion, physical restraint, and violations of human rights were common. In order to avoid such terrible conditions, reforms of psychiatric care systems were initiated in many Western countries. Psychiatrists and policy-makers developed planning principles for improving mental healthcare, such as [1, 2]:

- De-institutionalization as the basis for autonomy
- Healthcare provision close to the community
- Less inpatient care, more outpatient care
- Integration into general healthcare

For future planning, it is essential to assess the extent to which these principles have been implemented to date and to evaluate their suitability as guiding frameworks for forthcoming initiatives. Accordingly, we apply these four principles to systematically analyze past implementation efforts, identify existing shortcomings, and determine the actions required for future development.

## De-institutionalization

### Downsizing psychiatric hospitals and reducing beds

Old psychiatric hospitals were not only used for the treatment of acute psychiatric illness periods—they were also the place of long-term stays for individuals with severe and chronic mental illness. Similar to patients with somatic illnesses, some people in very acute mental states can be treated best as inpatients. Treatment in psychiatric wards should be limited to short periods of acute treatment, while hospitals should not serve as accommodation for chronic states. Thus, a major aim of the reforms was to promote a living situation for persons with severe mental illness that was independent of institutions by moving them out of large psychiatric hospitals (called “de-institutionalization”). For this reason, the number of psychiatric beds was reduced dramatically and psychiatric hospitals were downsized or even closed.

Policies for mental healthcare reform varied across European countries and often within countries. Some concepts focused on the development and rollout of outpatient clinics and outreach services for people living in their own households, while others preferred to substitute each hospital bed with a place in another service, such as in nursing homes or day hospitals [3]. For example, the Swiss canton of Bern had overall 2.22 beds or places per 1000 inhabitants in psychiatric hospital departments, day hospitals and, residential homes. Their strategy aimed to reduce psychiatric hospital beds from 0.96 to 0.40 per 1000 inhabitants. In parallel, they planned to increase the places in private hospitals, addiction hospitals, forensic hospitals, day hospitals, and sheltered housing from 1.26 to 1.82 per 1000 inhabitants [3]. Thus, the overall number of beds/places remained unchanged (2.22 beds/places per 1000 inhabitants). Some authors criticized this process as “trans-institutionalization,” because people with mental illness remained dependent on institutions.

Patients of day hospitals decide whether they attend this service. Thus, they have much more autonomy than psychiatric inpatients and there is no risk of physical restraint. However, living in a nursing home or a residential home is not equal to independent living. Regulations in such housing facilities are less restrictive compared to hospitals. Consequently, this increases independence and the chance for self-determination. Nevertheless, research on housing preferences showed that residents of psychiatric homes prefer to live in private households [4].

It has been argued that patients could learn social skills during their long stays in the old psychiatric hospitals, which is not possible today because of shorter inpatient treatment. These skills can now be learned and practiced in sheltered housing facilities or in private households offering more self-determination. In addition, people with mental illness clearly prefer to live in private households [4].

Several authors reported that a period of re-institutionalization started in the 1990s. The number of highly specialized psychiatric hospital beds increased, such as forensic beds, psychosomatic beds, or beds in rehabilitation hospitals [3, 5]. Despite the steep increase in forensic beds in several countries, the sum of psychiatric hospital and forensic beds decreased (Table 1).

**Table 1:** Reduction in general psychiatric beds and increase in forensic beds in selected Western European countries (rates per 1000 population [5]).

	England	Germany	Netherlands
*Psychiatric hospital beds*			
In 1990	1.318	1.417	1.592
In 2001	0.628	1.282	1.355
Change in %	−52%	−10%	−15%
*Forensic beds*			
In 1990	0.013	0.046	0.047
In 2002	0.018	0.078	0.114
Change in %	+38%	+70%	+143%
*Sum of psychiatric hospital beds and forensic beds*			
In 1990	1.331	1.463	1.639
In 2002	0.646	1.36	1.469
Changes in %	−51%	−7%	−11%

In addition, the number of places in sheltered housing increased during this period. Priebe at al. [5] reported that in several European countries the increase in the number of beds in psychiatric housing facilities and in forensic services was greater than the decrease in psychiatric hospital beds.

### Sheltered or independent housing

Frequently, residents of housing facilities have their own room in a building with shared facilities such as dining or living rooms, with services provided by staff. This type of sheltered housing is a compromise between independent living in the community and a residential institution [6]. The idea of this model was to provide more autonomous and less restrictive housing incrementally over time [4]. The overall goal of this step-by-step process was full integration into the community. Due to various reasons, this objective could not be reached for the large majority of residents. Thus, many persons with long-standing and severe mental illness remained in nursing homes or custodial settings. Among those who do not stay in homes, 20–70% are living in their parents’ household, which is considered another form of dependency [7].

Many people with severe mental illness who did not receive the necessary care lost their accommodation and became homeless. Studies have shown that high proportions of the homeless population have severe mental illness [7]. Being homeless worsens psychiatric prognosis and prevents recovery, resulting in more chronic and disabling illness courses. This situation is more pronounced in North America than in Europe.

In the last 15 years, several randomized controlled trials had been conducted investigating the Housing First concept among homeless people with mental illness [4]. Many traditional concepts of housing rehabilitation started with psychiatric treatment before considering accommodation. The Housing First concept gives higher priority to independent housing than to treatment adherence. This concept is based on the assumption that living in normal accommodation will help to improve psychosocial functioning and independent living. Further, a clear separation of accommodation from psychiatric and medical service provision is recommended. Following this idea, the accommodation must be rented by patients themselves and not by psychiatric or social agencies. Provision of professional support is flexible and considers the patient’s actual needs. This approach gives persons with severe and persisting mental illness more autonomy and individual responsibility than all other housing options [4].

Recently, a randomized controlled study from France reported 4‑year follow-up findings among homeless people with mental illness [8]. Those in the Housing First program showed more autonomy, higher housing stability, fewer inpatient days, and better quality of life. Despite higher alcohol consumption in the intervention group, substance dependence and psychiatric symptoms did not differ between groups. A systematic review of randomized controlled trials among homeless reported similar findings [4]. Regarding non-homeless people, the data on the effectiveness of Housing First programs are less clear. An initial pilot study in Switzerland showed that after 12 months almost all participants in the intervention group were able to live independently and the frequency of psychiatric inpatient treatment could be reduced significantly [9]. No significant differences were found regarding social inclusion and clinical outcomes such as quality of life, needs, mental status, or functioning. While Housing First was confirmed to be effective among homeless people with mental illness, the findings were less clear regarding non-homeless people with mental illness.

## Healthcare provision close to the community

For many decades, psychiatric inpatient care was located in psychiatric hospitals in the countryside far from cities and other communities. The influence of distance on the use or nonuse of psychiatric services was described more than 150 years (“Jarvis’s law” [1]): People living close to psychiatric hospitals show a higher chance of being admitted than people living far away. In the last 50 years, numerous studies confirmed the inverse relationship between the distance to psychiatric hospitals and admission rates. Similar findings were reported regarding the utilization of psychiatric outpatient services. This inverse relationship was confirmed when using several criteria for distance: road kilometers, travel time by car, and travel time using public transportation. Meise and colleagues [1] reported that a travel time of more than 30 min reduced the frequency of contacts with psychiatric inpatient and outpatient services. Such studies led to the recommendation that psychiatric services should be close to the community in which people with mental illness live.

The long distances between old psychiatric hospitals and the patients’ place of residence limited contacts with family members and friends. The decentralization of psychiatric inpatient care by creating psychiatric departments in general hospitals reduced the travel time for family members and friends visiting patients in hospital. Further, the short travel time to the patient’s home made it possible for patients to engage in activities outside the hospital, even during more acute treatment episodes. Implementing psychiatric inpatient care in decentralized general hospitals is a very long process, sometimes requiring more than 20 years [10], and in many Western countries it is not yet complete.

## Integration of psychiatric services into general healthcare

Five decades ago, Goldberg and Huxley [11] published their famous book *Mental Illness in the Community*, reporting that nearly all people with mental illness contact their primary care physicians. Less than two thirds of primary care patients with psychiatric illness were identified by their doctors as having mental disorders. The large majority (seven out of eight) of those identified were treated by primary care physicians themselves. Only one eighth were referred to a mental health specialist. Thus, primary care physicians are the medical core group responsible for appropriate diagnosis and treatment of people with mental illness.

Other studies reported that many inpatients of non-psychiatric hospital wards have coexisting psychiatric disorders. Point-prevalence estimates for any psychiatric disorder have been reported to range between 20% and nearly 40% [12]. Similar to primary care, non-psychiatric physicians often failed to recognize psychiatric disorders. As a result, many patients with mental illness did not receive any treatment for their condition. These comorbid mental disorders have negative consequences such as delayed recovery, prolonged inpatient treatment, and increased risk of chronic disability [13]. The fact that in some non-psychiatric hospital wards the psychiatric prevalence was higher in rural catchment areas than in urban areas led to the hypothesis that the travel time between the place of residence and the nearest psychiatric inpatient unit could be the reason for this difference [1]: People with mental illness who live far from the next psychiatric inpatient unit may have had an increased risk of being admitted to non-psychiatric hospital wards.

In addition, individuals with mental illness have a shorter life expectancy compared to those who do not have such conditions. This increased mortality among persons with psychiatric disorders is often caused by physical comorbidity. For example, persons with schizophrenia or affective disorders more often have cardiovascular diseases, diabetes, hepatitis, and respiratory tract diseases than the general population. Studies showed that persons with severe mental illness often do not receive the necessary somatic examination and treatment. The separation of psychiatric services from somatic medical services has been reported as a reason for the poor physical health outcomes of those with severe mental illness.

In many countries, these studies triggered more comprehensive training on mental health for undergraduate medical students and the inclusion of psychiatry into the curricula for general practitioners. The treatment gap in non-psychiatric hospital departments resulted in the proposal to include psychiatric services in general hospitals [1]. Treating psychiatric inpatients in decentralized general hospitals instead of central psychiatric hospitals has an important advantage: The provision of somatic and psychiatric expertise in the same hospital improves recognition and treatment of mental and physical disorders in psychiatric as well as in non-psychiatric wards. As mentioned earlier, the implementation of psychiatric wards in general hospitals has taken many years [10], and in many countries it is not yet complete [13].

## Less inpatient care, more outpatient care

In order to provide appropriate psychiatric care, a variety of outpatient services were developed including outpatient clinics, case management services, assertive community treatment teams, and crisis intervention services. In the last four decades, numerous sophisticated studies have been conducted analyzing the effects of these services on people with severe and longstanding mental illness.

For example, several randomized controlled trials investigated assertive community treatment, which was developed for the outpatient treatment of those who had previously lived in psychiatric hospitals. A meta-analysis [14] showed that assertive community treatment reduced the likelihood of being admitted to hospitals and shortened the time spent in psychiatric hospitals. Patients receiving this intervention were more likely to stay in contact with psychiatric services compared to those receiving usual care. Further, significant positive effects on accommodation and employment were shown.

Unfortunately, such services, which have been shown to be effective, are often not provided at all or only insufficiently provided. Reasons for this situation are cuts in budgets, inadequate reimbursement for specific treatments, privatization of health services, and changes in the structures of mental healthcare services. Thus, often the provision of such services for people with longstanding and severe mental disorders is deficient and inadequate [15]. In addition, the shortage of qualified and specialized mental healthcare personnel is pressing in several countries [16].

## Learning from past decades

Planning for the future requires analyses of the achievements, disadvantages, and limitations of recent health service development and implementation. The most important aim of the reforms was to avoid violations of human rights in psychiatric hospitals and to facilitate independent living in the community, also for those with the most severe illness [1, 2]. For this purpose, it was requested to reduce the number of psychiatric hospital beds. Even though the number of psychiatric beds has increased recently, the overall number of beds in psychiatric hospitals has decreased dramatically over the last five decades. Given this fact, the mental healthcare reforms may be considered to have been implemented successfully.

In many European countries the number of forensic beds increased substantially. Admissions to forensic psychiatry frequently involve prolonged inpatient treatment and have various other negative effects. But what are the reasons for more forensic beds? It was discussed whether the reduction in general psychiatry beds was compensated for by an increase in forensic beds; that is, whether there is a causal association. Some authors note that there are no indicators that the number of homicides, assaults, and other crimes committed by people with mental illness has increased [5]. We know that changes in legislation and in the interpretation of laws occurred. Overall, it is important to understand the reasons for the higher number of people with mental illness in forensic psychiatry. This requires focused and comprehensive research.

Many people with longstanding and severe mental disorders live in sheltered facilities. Despite the fact that sheltered housing is a compromise, this is an enormous advantage and allows for significantly more autonomy than 50 years ago. Persons with severe mental illness show an increased risk for homelessness. The Housing First studies among homeless found clear positive effects on autonomy, housing stability, reduced hospital admissions, and quality of life. In many Western countries, more services providing Housing First for homeless people with mental illness are needed. Unfortunately, studies investigating this promising approach among non-homeless are scarce.

Many others live in their parents’ household and receive support from their families. Often this results in less leisure time, high financial expenses, and other relevant burden for the families. Studies have shown that families often do not receive the necessary support for these caring tasks [17]. Policy-makers must provide more social and psychiatric support for family caregivers.

In the last few decades, psychiatric units for inpatient treatment were established in general hospitals alongside the downsizing of stand-alone psychiatric hospitals [10]. This improves the collaboration between psychiatrists and specialists in somatic medicine. Unfortunately, this process of decentralization took a long time. Nevertheless, it must be accelerated. Despite the fact that psychiatric training for primary care doctors and other physicians improved in the last few decades, many persons with somatic–psychiatric comorbidity are overlooked and do not receive appropriate treatment. Innovative concepts have to be developed, evaluated, and implemented in order to solve this enormous problem [13].

In the last few decades, various services for psychiatric outpatient care have been established. Unfortunately, specialized services that have been shown to be most effective (e.g., assertive community treatment) are often not available or only insufficiently provided. Despite financial constraints, policy-makers must find ways to provide the necessary services.

## The context of future mental health service planning

Planning and implementing health services are influenced by major societal developments and the political climate. Five decades ago, democracy, solidarity, and freedom from authoritarian structures were shared by the majority of the population. These ideas supported concepts such as de-institutionalization, human rights, inclusion, and independent living.

In recent years, several societal trends have changed. More and more people favor the exclusion of people who are perceived as “different” such as migrants, persons with severe mental illness, or marginalized groups [18]. Increasing stigmatization of persons with schizophrenia is among the results of such developments. Violence against disadvantaged groups, dissident individuals, or political opponents occurs more frequently. Violent attacks on medical staff (in all medical specialties, not only in psychiatry) are being reported with increasing frequency.

Governmental budgets for social affairs have been cut in many communities. Thus, budgets for mental health services or family self-help groups are also frequently cut. Non-profit engagement will not be able to compensate for this. Shortage of affordable apartments often results in more difficult housing rehabilitation. As a result, a growing number of people with severe mental illness depend on sheltered housing facilities, and the risk for homelessness increases.

The aforementioned societal changes occurred in many Western countries, and these developments may pose major difficulties for implementing better mental healthcare [18]. However, we must take this new societal reality into account and find innovative solutions to improve psychiatric care under these circumstances.
